# Folic acid protects against isoniazid-induced liver injury via the m^6^A RNA methylation of cytochrome P450 2E1 in mice

**DOI:** 10.3389/fnut.2024.1389684

**Published:** 2024-05-10

**Authors:** Lan Jiang, Ya Ni, Cong Zhao, Dexin Gao, Xiaochun Gai, Ke Xiong, Jinyu Wang

**Affiliations:** Institute of Nutrition and Health, School of Public Health, Qingdao University, Qingdao, China

**Keywords:** CYP2E1, folic acid, RNA methylation, tuberculosis-induced liver injury, S-adenosyl methionine, S-adenosyl homocysteine

## Abstract

**Background:**

Cytochrome P450 2E1 (CYP2E1) converts isoniazid (INH) to toxic metabolites and is critical in INH-induced liver injury. The aim is to investigate the effect of folic acid (FA) on CYP2E1 and INH-induced liver injury.

**Methods:**

Male Balb/c mice were used. The mice in the control group only received an AIN-93M diet. The AIN-93M diet was supplemented with 0.66 g INH/kg diet for the mice in the INH and FA groups. The mice in the FA group were treated with additional 0.01 g FA/kg diet. The one-carbon cycle metabolites, the expressions of CYP2E1 and the DNA and RNA methylation levels were detected to reveal the potential mechanism.

**Results:**

FA treatment significantly reduced the alanine aminotransferase level and alleviated the liver necrosis. The mRNA and protein expressions of CYP2E1 were significantly lower in the FA group than those in the INH group. The *N*^6^-methyladenosine RNA methylation level of *Cyp2e1* significantly increased in the FA group compared with the INH group, while the DNA methylation levels of *Cyp2e1* were similar between groups. Additionally, the liver S-adenosyl methionine (SAM)/S-adenosyl homocysteine (SAH) was elevated in the FA group and tended to be positively correlated with the RNA methylation level of *Cyp2e1*.

**Conclusion:**

FA alleviated INH-induced liver injury which was potentially attributed to its inhibitory effect on CYP2E1 expressions through enhancing liver SAM/SAH and RNA methylation.

## 1 Introduction

During anti-tuberculosis treatment, 5–33% patients suffered from drug-induced liver injury (1). Isoniazid (INH), the major first-line anti-tuberculosis drug, is known to induce liver injury during anti-tuberculosis treatment (1, 2). Liver injury hampers anti-tuberculosis therapy, lowers recovery rate, and contributes to high mortality (3, 4). However, no effective treatment exists.

Folic acid (FA) supplementation has been demonstrated to be beneficial for various liver diseases or injuries, such as alcoholic liver disease (5), nonalcoholic fatty liver disease (6), and liver injuries caused by external agents (7, 8), primarily due to its anti-inflammatory and antioxidant attributes. A cross-sectional study of 3,302 patients found that FA supplementation was a protective factor against INH-induced liver injury (9). Our previous study indicated that FA can protect against INH/rifampicin-induced liver injury in rats (10), however, the detailed mechanism is largely unknown.

Cytochrome P450 2E1 (CYP2E1), the major cytochrome P450 enzyme in the liver, plays a critical role in INH-induced liver injury (1). CYP2E1 catalyzes the conversion of INH to diazo hydrides and reactive oxygen species which are toxic to the liver (11–14). A meta-analysis including 1,625 individuals found that people with a high CYP2E1 activity had an increased risk of tuberculosis-drug-induced liver injury compared to those with a low CYP2E1 activity (OR: 1.4, 95% CI: 1.1–1.8) (15). Consistently, in a rat experiment, the use of a CYP2E1 inhibitor, diallyl sulfide, was demonstrated to alleviate INH-induced hepatotoxicity (16). However, the factors that regulate the expression and activity of CYP2E1 are not well-understood.

A recent animal study showed that the expressions of CYP2E1 may be related to the DNA methylation of the *Cyp2e1* gene (17). In addition, supplementation of S-adenosyl methionine (SAM), a methyl donor for DNA or RNA methylation, was shown to reduce the activity of CYP2E1 and alleviate oxidative liver injury in micropigs fed with a folate-deficient diet plus ethanol (18). These nascent evidence points to the direction that CYP2E1 may be regulated by DNA or RNA methylation.

FA participants in one-carbon cycle and provides methyl groups for DNA, RNA or protein methylation (19). Folate-deficient diet was shown to perturb the one-carbon cycle, decrease the global DNA methylation level and aggravate ethanol-induced liver injury (20). We hypothesize that FA may alleviate INH-induced liver injury by downregulating CYP2E1 expressions through regulating DNA or RNA methylation. In this study, the effect of FA supplementation on INH-induced liver injury is investigated in mice. The one-carbon cycle metabolites, the expressions of CYP2E1 and the DNA and RNA methylation levels are determined to reveal the potential mechanism.

## 2 Methods

### 2.1 Ethics

All animal protocols adhered to the US National Research Council’s Guide for the Care and Use of Laboratory Animals (8th edition). The protocols were approved by the Experimental Animal Welfare Ethics Committee of Qingdao University (No. 20201107Balb/c3020210118048).

### 2.2 Animal experiment

Male Balb/c mice (eight-week-old) weighing 23.4 ± 2.2 g were obtained from Beijing Vital River Laboratory Animal Technology Co., Ltd. (Beijing, China). Mice had free access to water and food, and were maintained at an ambient temperature of 21 ± 2°C, a humidity ranging from 45 to 65% under a 12 h light/dark cycle.

After one-week adaptive feeding by a standard AIN-93 M diet, the mice were randomly assigned into three groups: the control group (CON, *n* = 7), the INH group (*n* = 7) and the FA group (*n* = 8). Mice were fasted overnight and humanely euthanized after a 72-day intervention. Serum was separated after centrifugation at 3500 rpm for 15 min at room temperature. The body weight of the mice was weighed every 2 days. Liver tissues were fixed in a 4% paraformaldehyde solution and embedded in paraffin for histological analysis. The rest of liver tissues were immediately frozen in liquid nitrogen and then stored at −80°C.

### 2.3 Dosage information

The CON group received an AIN-93M diet. The INH group received an AIN-93M diet supplemented with 0.66 g INH/kg diet. The FA group received additional 0.01 g FA/kg diet based on the INH diet. The dosages of INH and FA were referenced to previous studies (10, 21). INH (MB1501-1, purity, >99%) and FA (MB1501-1, purity, >98%) were purchased from Meilun Biotechnology Co., Ltd. (Dalian, China).

### 2.4 Biochemical analyses

The alanine aminotransferase (ALT, JM-03154M1) and aspartate transaminase (AST, JM-03113M1) were measured using commercial kits from Jingmei Biotechnology Co., Ltd. (Jiangsu, China). The γ-glutamyltransferase (γGT, YX-W-A208) was measured using commercial kits from Sinobest Biotechnology Co., Ltd. (Shanghai, China) and the malondialdehyde (MDA, S0131S) was measured using commercial kits from Beyotime Biotechnology Co., Ltd. (Shanghai, China). The SAM (RF8761) and SAH (RF8767) were analyzed via commercial kits from Ruifan Biotechnology Co., Ltd. (Shanghai, China).

### 2.5 Liver histological analysis

Paraformaldehyde-fixed, paraffin-embedded liver tissues were sectioned at 5 μm thickness. The liver tissues were stained with hematoxylin and eosin (H&E). Images of the liver sections were acquired using a light microscope. Histology Activity Index (HAI-Knodell score) was used to grade the pathological changes of liver (22).

### 2.6 Western blotting

The western blotting was carried out based on previous reports (10, 23). The total protein was extracted from the liver tissues and the concentration was measured via a bicinchoninic acid assay kit (ZJ101, Epizyme Biomedical Technology Co., China). The proteins were separated by gel electrophoresis and transferred to a polyvinylidene fluoride membrane. The membrane was blocked and incubated with primary antibodies against CYP2E1 (ab28146, Abcam, Cambridge, United Kingdom) and β-Actin (ab8227, Abcam, Cambridge, United Kingdom) overnight at 4°C. Then horseradish peroxidase-conjugated secondary antibodies (ab205718, Abcam, Cambridge, United Kingdom) were used to bond with primary antibodies for 1 h at room temperature. The protein levels were visualized by Fusion Solo S imaging system (Vilber Co., France) and quantified by ImageJ program (National Institutes of Health, United States). Each panel had samples with pooled quality control (QC) and the intensity of each protein was normalized by the intensity of β-actin and QC.

### 2.7 Real-time polymerase chain reaction (real-time PCR)

The procedure of real-time polymerase chain reaction (real-time PCR) was carried out according to the previous study (10). Total RNA was extracted from the liver tissues using Trizol reagent (R411, Vazyme Biotech Co., Nanjing, China) according to the manufacturer’s protocol. RNA was quantified with a Nanodrop spectrophotometer and reverse transcription was performed using HiScript III RT SuperMix (Vazyme Biotech Co., Nanjing, China). The primers of *Cyp2e1*, *Tnfa*, *Il6*, *Il1b*, *F4/80* and *Gapdh* are listed in Table 1. RT-PCR was performed in a Bio-Rad CFX96 Touch real-time PCR system (Bio-Rad Laboratories, United States) with *Gapdh* as the internal control. The expression of genes was determined by the ΔΔCT method.

**Table 1:** Primer sequences used for RT-PCR.

Target genes		Sequences
*Cyp2e1*	Forward	5′-GCATCCAAAGAGAGGCACACTTCC-3′
	Reverse	5′-GCACAGCCAATCAGAAAGGTAGGG-3′
*Tnfa*	Forward	5′-CACCACGCTCTTCTGTCTACTGAAC-3′
	Reverse	5′-AGATGATCTGAGTGTGAGGGTCTGG-3′
*Il6*	Forward	5′-CTTCTTGGGACTGATGCTGGTGAC-3′
	Reverse	5′-TCTGTTGGGAGTGGTATCCTCTGTG-3′
*Il1b*	Forward	5′-CACTACAGGCTCCGAGATGAACAAC-3′
	Reverse	5′-TGTCGTTGCTTGGTTCTCCTTGTAC-3′
*F4/80*	Forward	5′-TTCCTGCTGTGTCGTGCTGTTC-3′
	Reverse	5′-GCCGTCTGGTTGTCAGTCTTGTC-3′
*Gapdh*	Forward	5′-GACATGCCGCCTGGAGAAAC-3′
	Reverse	5′-AGCCCAGGATGCCCTTTAGT-3′

### 2.8 DNA and RNA methylation

The 5-methylcytosine (5mC) DNA and *N*^6^-methyladenosine (m^6^A) RNA methylation were measured in the liver. The transcription start site (−5,000 bp to +1,000 bp) sequence of *Cyp2e1* gene was obtained from NCBI^1^ to predict the methylation sites. MassArray technology was performed to investigate the methylation of CpG site in the promoter region of *Cyp2e1* (24). DNA was extracted from the liver tissues. Commercial NaHSO3 kits (Zymo Research Biotech Co., California, United States) was used to deaminate the non-methylated cytosine (C) and convert it to uracil (U). The gene fragments were enriched and amplified by PCR reactions, and the products were treated with shrimp alkaline phosphatase to remove free dNTPs from the system. The small fragment containing CpG site was obtained after simultaneously reverse transcribing and digesting with T7 RNA & DNA Polymerase and RNaseA enzymes. The purified product was transferred to a SpectroCHIP^®^ bioarray using an Agena NanodispenserRS1000 (Agena Bioscience, California, United States) spotting instrument. The spotted SpectroCHIP chips (Agena Bioscience, California, United States) were analyzed using MALDI-TOF (Agena Bioscience, California, United States) and the results were obtained by an EpiTYPER^™^ software (Agena Bioscience, California, United States).

The RNA methylation were analyzed according to previously published protocol (25). Briefly, the extracted RNA, nuclease-free water, 5 × IP buffer, and RNAase inhibitor were configured to form the RNA Binding Protein Immunoprecipitation (RIP) system. The RIP system was mixed with pre-conjugated m^6^A antibody (A17924, ABclonal, Wuhan, China)-Protein A/G magnetic beads (RM02915, ABclonal, Wuhan, China) and then incubated for 1 h at 4°C. Magnetic beads were adsorbed using a magnetic holder and washed twice. Elution buffer was added to the beads and incubated at 50°C for 1.5 h. The supernatant was collected to extract RNA. The enrichment of methylated *Cyp2e1* was detected using real-time PCR.

### 2.9 Statistical analysis

The data were expressed as mean ± standard deviation (SD). Difference among the three groups was analyzed by a one-way analysis of variance test. Post-hoc pairwise comparisons were adjusted by the least significant difference method. Pearson correlation analysis was employed to examine the associations between variables including one-carbon metabolites, protein and RNA expression of CYP2E1, DNA and RNA methylation of *Cyp2e1*, and liver function indicators. Statistical analyses were performed by SPSS, version 26 (IBM, New York, United States). Outliers, more than three SDs from the mean, were removed. *p* < 0.05 was considered statistically significant. GraphPad Prism version 8 (GraphPad Software Inc., CA, United States) and Adobe Illustrator 2020 (Adobe Inc., CA, United States) were used for graphing.

## 3 Results

### 3.1 Effects of FA treatment on INH-induced liver injury

**Figure 1:**
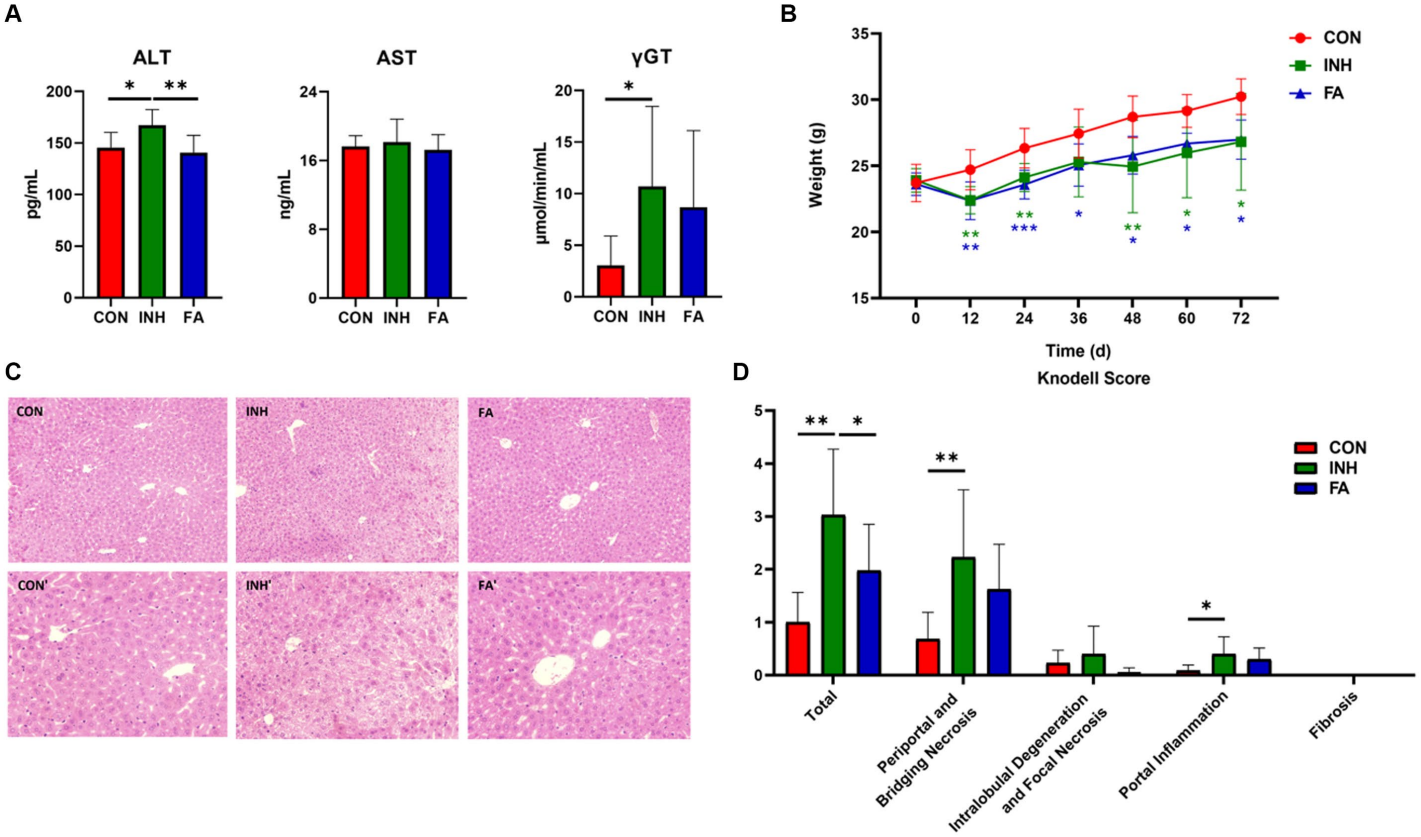
FA treatment improved liver injury in INH-fed mice. **(A)** Serum ALT, AST and γGT levels. **(B)** Change of body weight during the 72-day experiment. **(C)** Representative images of liver specimens stained with hematoxylin and eosin (magnification ×200 or ×400) (CON, ×200; CON′, ×400; INH, ×200; INH′, ×400; FA, ×200; FA′, ×400). **(D)** Liver toxicity score. The sample size was 7, 7 and 8 in the CON, INH and FA group, respectively. ^*^p < 0.05, ^**^p < 0.01, and ^***^p < 0.001. CON, control; INH, isoniazid; FA, folic acid; ALT, alanine aminotransferase; AST, aspartate aminotransferase; γGT, gamma-glutamyl transferase.

As shown in Figure 1A, the serum ALT and γGT were significantly higher in the INH group than those in the CON group; the serum AST tended to increase in the INH group compared with the CON group, though the difference was not statistically significant. FA treatment significantly reduced the serum ALT and tended to reduce the serum AST and γGT compared with the INH group. At the end of the experiment, the body weight of the mice in the INH and FA groups was significantly lower than that in the CON group (Figure 1B). The histopathological effects of INH and FA treatment on liver tissues were shown in Figures 1C,D. Mice in the CON group showed well-arranged hepatic cords in a radial pattern. In the INH group, the structure of liver lobules was impaired by various types of necrosis such as piecemeal necrosis, bridging necrosis, and focal necrosis and infiltration of inflammatory cells. FA treatment partially restored the liver lobule structure. In the FA group, the cytoplasmic staining was uniform, and the nucleus was clear. The liver toxicity score was significantly elevated in the INH group compared with the CON group, and the score was significantly lower in the FA group than that in the INH group.

### 3.2 Effects of FA on oxidative stress and inflammation levels

**Figure 2:**
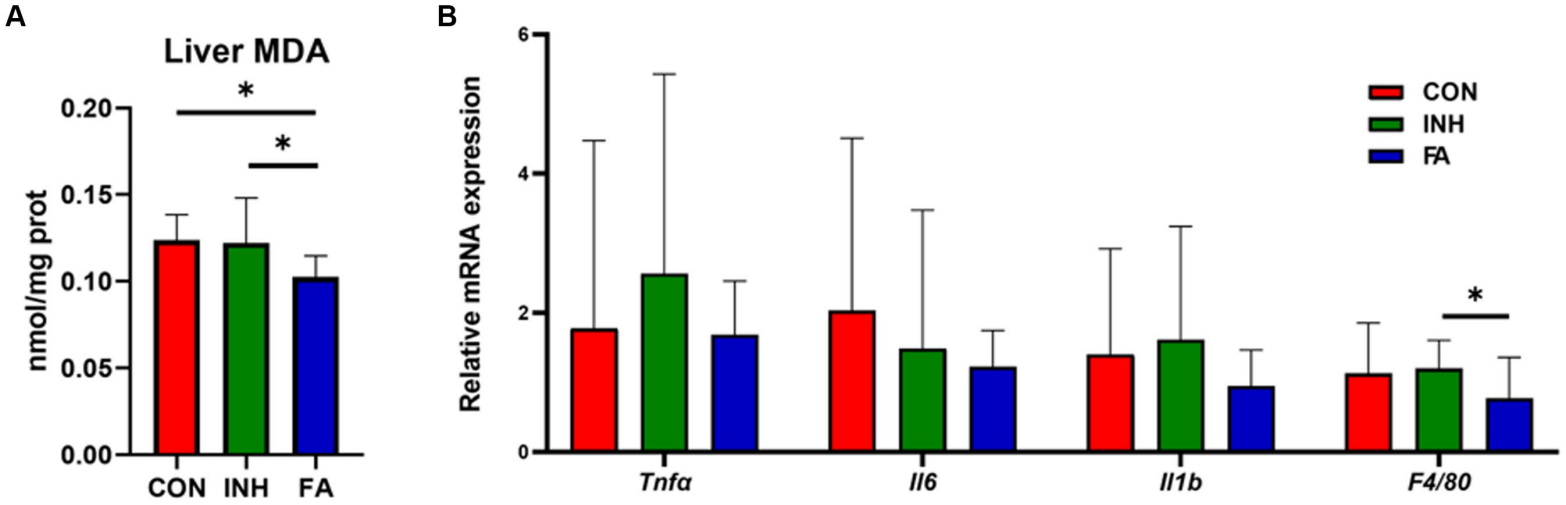
The expression levels of oxidative stress and inflammation. **(A)** Liver MDA. **(B)** Inflammation-related genes. The sample size was 7, 7 and 8 in the CON, INH and FA group, respectively. ^*^p < 0.05. CON, control; INH, isoniazid; FA, folic acid; MDA, malondialdehyde; *Tnf*α: tumor necrosis factor-α; *Il6*: interleukin-6; *Il1b*: interleukin-1β; *F4/80*: mouse EGF-like module-containing mucin-like hormone receptor-like 1.

The liver MDA level was significantly lower in the FA group than that in the INH group (Figure 2A). The mRNA levels of *Tnfa*, *Il1b*, and *F4/80* tended to be higher in the INH group than that in the CON group (Figure 2B). FA treatment significantly decreased the mRNA level of *F4/80* and tended to decrease the mRNA levels of *Tnfa*, *Il6* and *Il1b* compared with the INH group.

### 3.3 Effects of FA on CYP2E1 expressions

**Figure 3:**
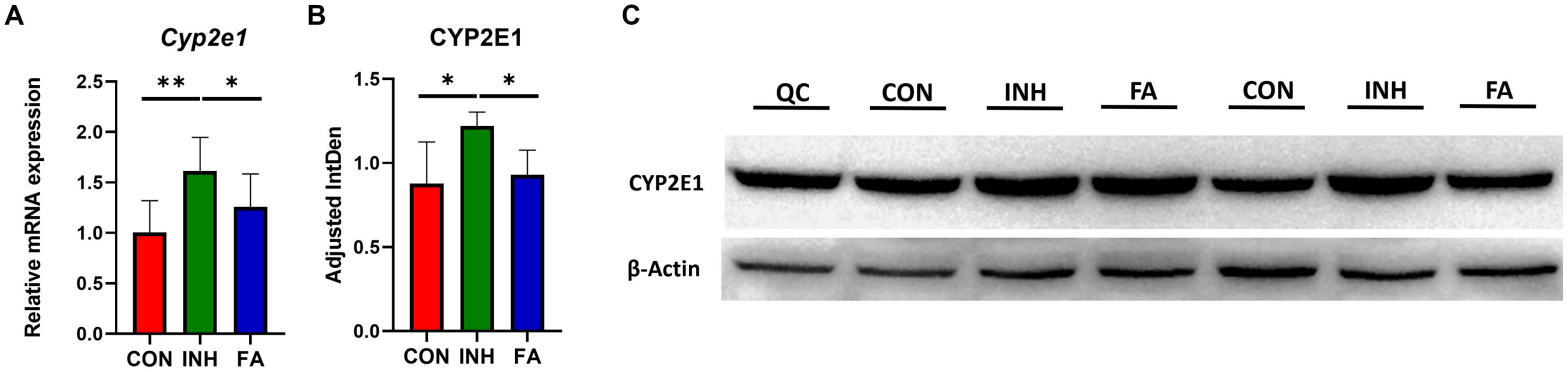
The mRNA and protein expression levels of CYP2E1. **(A)** Relative mRNA expression. The sample size was 7, 7 and 8 in the CON, INH and FA group, respectively. **(B)** Densitometric analysis of the protein expressions and **(C)** western blotting image (*N* = 4 in the CON, INH and FA group). ^*^p < 0.05 and ^**^p < 0.01. CON, control; INH, isoniazid; FA, folic acid; Mar, protein marker; CYP2E1, cytochrome P4502E1; QC: quality control.

The mRNA level of *Cyp2e1* was significantly elevated in the INH group but decreased following FA treatment (Figure 3A). Consistently, the protein level of CYP2E1 was significantly increased in the livers of INH mice compared with CON mice, which was significantly reduced after FA treatment (Figures 3B,C).

### 3.4 Effects of FA on SAM and SAH

**Figure 4:**
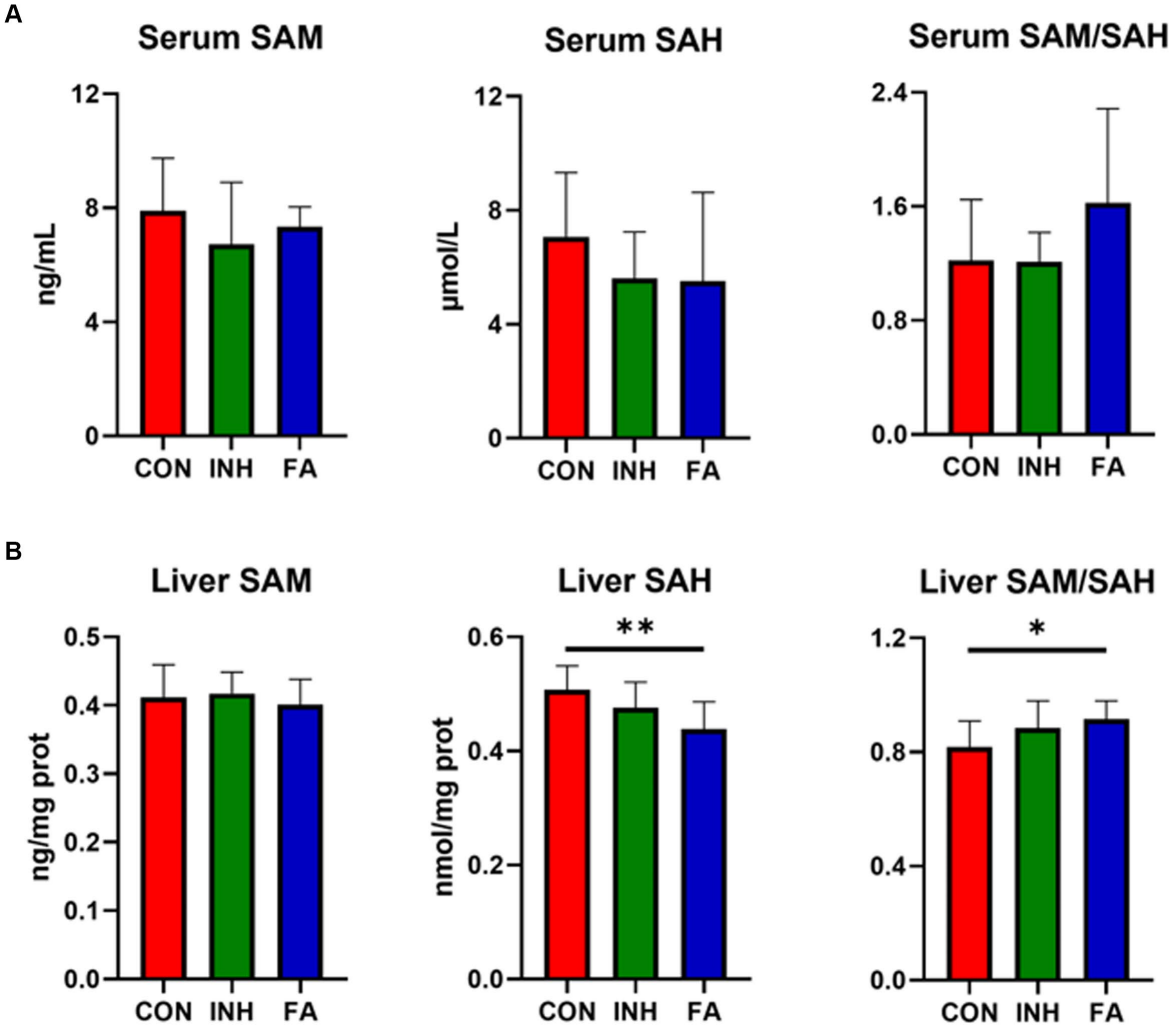
SAM and SAH level in the liver and serum. **(A)** SAM and SAH level in the serum. **(B)** SAM and SAH in the liver. The sample size was 7, 7 and 8 in the CON, INH and FA group, respectively. ^*^p < 0.05 and ^**^p < 0.01. CON, control; INH, isoniazid; FA, folic acid; SAM, S-adenosyl methionine; SAH, S-adenosyl homocysteine.

There was no significant difference for the serum SAM, SAH or SAM/SAH among the three groups, while an upward trend of serum SAM/SAH was observed in the FA group (Figure 4A). The liver SAM level was similar among the three groups (Figure 4B). While the liver SAH significantly decreased and liver SAM/SAH significantly increased in the FA group.

### 3.5 Effect of FA on *Cyp2e1* methylation

**Figure 5:**
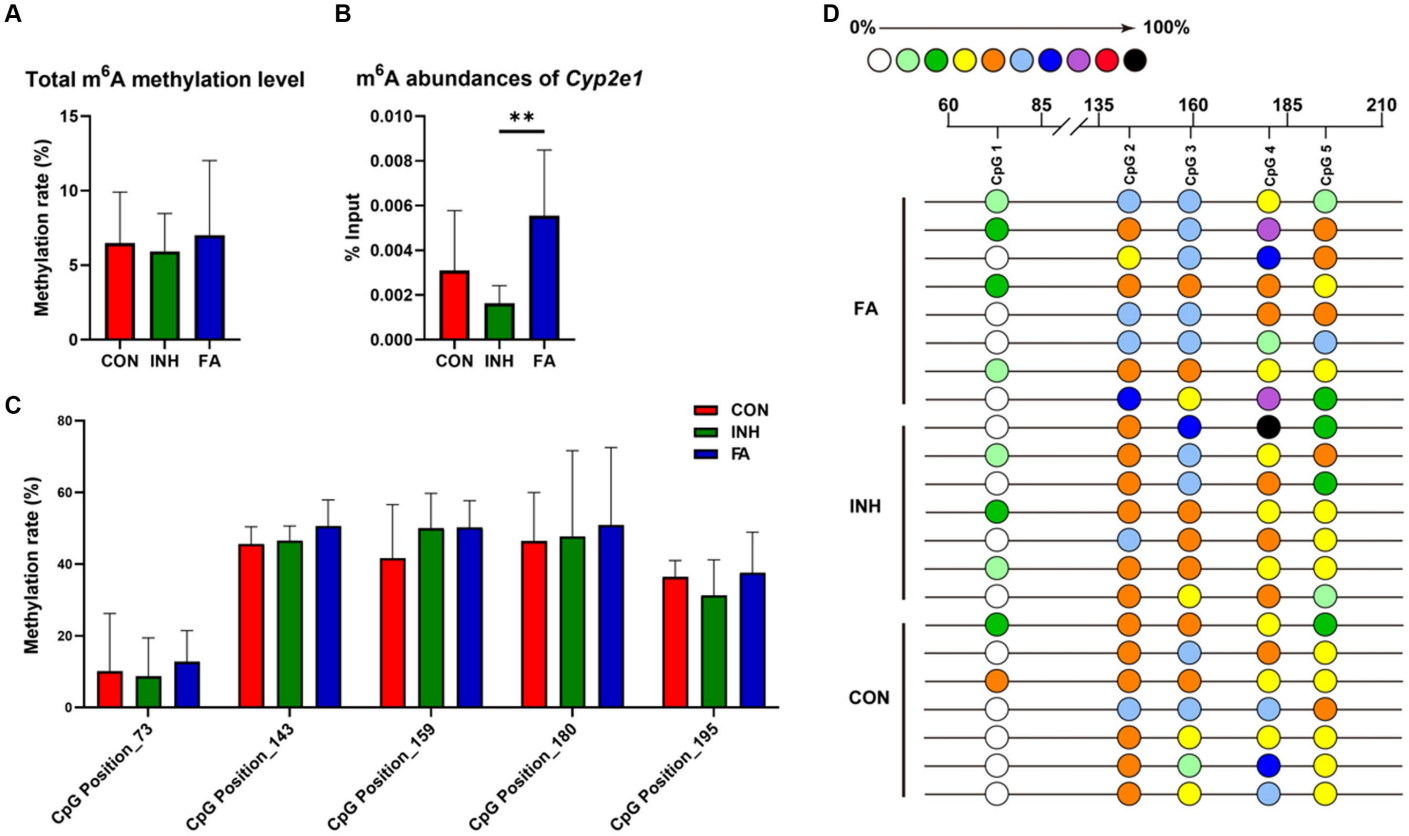
DNA and RNA methylation levels of *Cyp2e1*. **(A)** Total m^6^A methylation level. **(B)** m^6^A abundances of *Cyp2e1*. **(C)** MassARRAY quantitative DNA methylation analysis of CpG sites in *Cyp2e1*. **(D)** DNA methylation bubble map for each sample. Colored bubbles represent the methylation status of detected CpG sites in *Cyp2e1*. The sample size was 7, 7 and 8 in the CON, INH and FA group, respectively. ^**^p < 0.01. CON, control; INH, isoniazid; FA, folic acid; *Cyp2e1*, cytochrome P450 2E1; m^6^A, *N*^6^-methyladenosine.

For RNA methylation, the total m^6^A methylation level was similar between the CON and INH group and showed an upward trend in the FA group (Figure 5A). Gene-specific m^6^A-qPCR was performed to measure the RNA methylation level of *Cyp2e1*. The m^6^A abundances of *Cyp2e1* tended to decrease after INH treatment and markedly increased after FA treatment (Figure 5B). For DNA methylation, five CpG sites on the *Cyp2e1* gene were analyzed. No significant difference was observed for the 5mC DNA methylation level among the three groups (Figures 5C,D).

### 3.6 Correlations among one-carbon metabolites, CYP2E1, DNA or RNA methylation and liver function indicators

**Figure 6:**
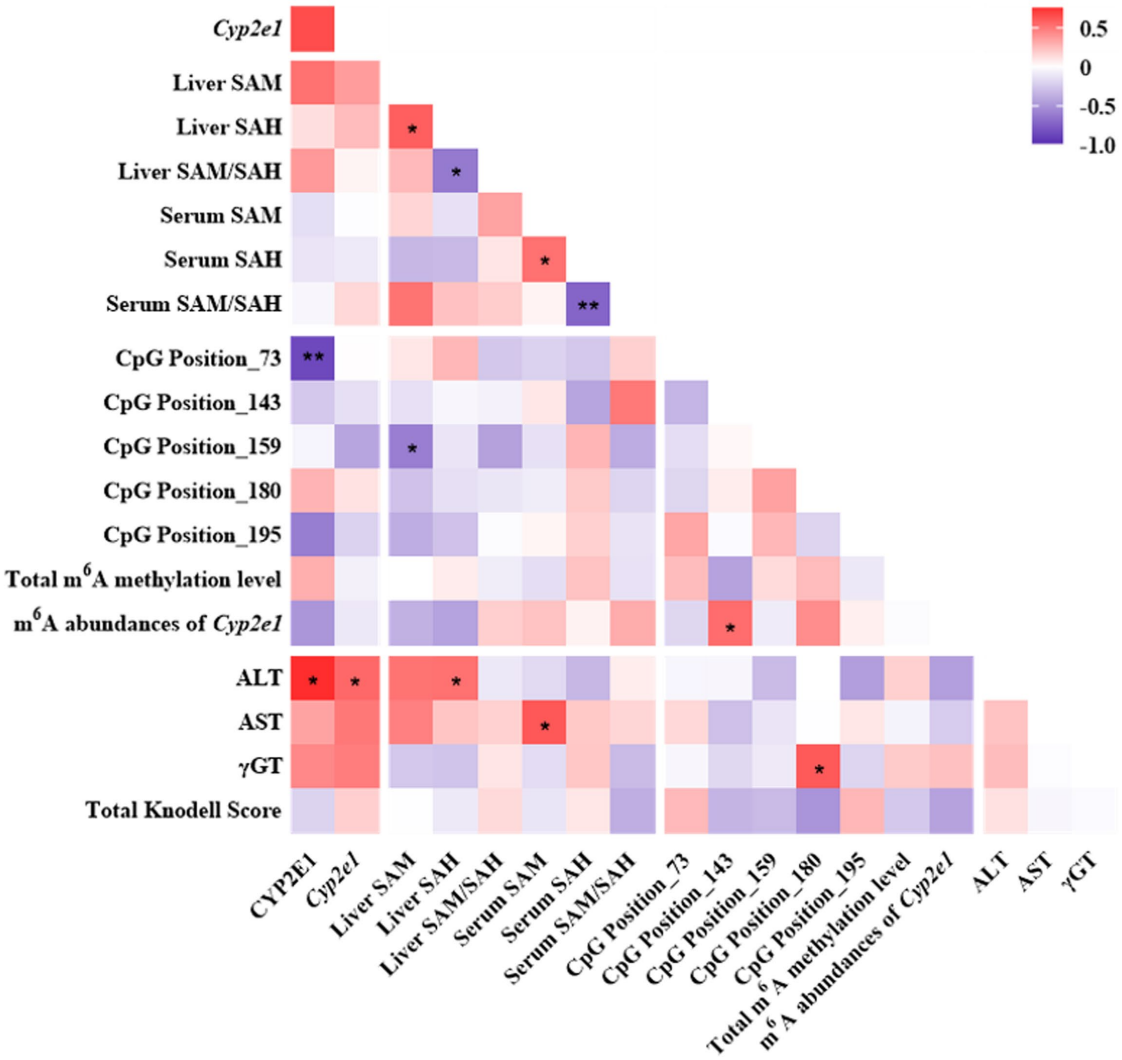
Pearson correlation analyses among one-carbon metabolites, CYP2E1, DNA or RNA methylation and liver function indicators in the INH and FA group. Purple and red colors indicate negative and positive correlations, respectively. The strength of the Pearson correlation directly correlates with the color intensity. ^*^p < 0.05 and ^**^p < 0.01. INH, isoniazid; FA, folic acid; CYP2E1, cytochrome P450 2E1; SAM, S-adenosyl methionine; SAH, S-adenosyl homocysteine; m^6^A, *N*^6^-methyladenosine; ALT, alanine aminotransferase; AST, aspartate aminotransferase; γGT, gamma-glutamyl transferase.

The mRNA and protein expressions of CYP2E1 were positively associated with ALT and tended to be positively associated with AST and γGT (Figure 6). The expression of CYP2E1 was negatively correlated with the methylation level of CpG Position 73 and tended to be negatively correlated with m^6^A RNA methylation level of *Cyp2e1*. The liver and serum ratio of SAM/SAH tend to be positively associated with the m^6^A abundances of *Cyp2e1*.

## 4 Discussion

Our results highlight the complex interplay among FA, RNA methylation, and CYP2E1 expression, which appears to be a key factor in alleviating INH-induced liver injury. To our knowledge, this is the one of the first studies to explore the effects of FA on the DNA and RNA methylation and expression of CYP2E1 and investigate CYP2E1 as a potential mechanism behind FA’s protection on INH-induced liver injury. Specifically, the observed results confirmed the protective effect of FA on INH-induced liver injury and indicated that this protective effect of FA may be related to its inhibition on CYP2E1 expression via enhancing the liver SAM/SAH ratio and the m^6^A RNA methylation of *Cyp2e1*.

**Figure 7:**
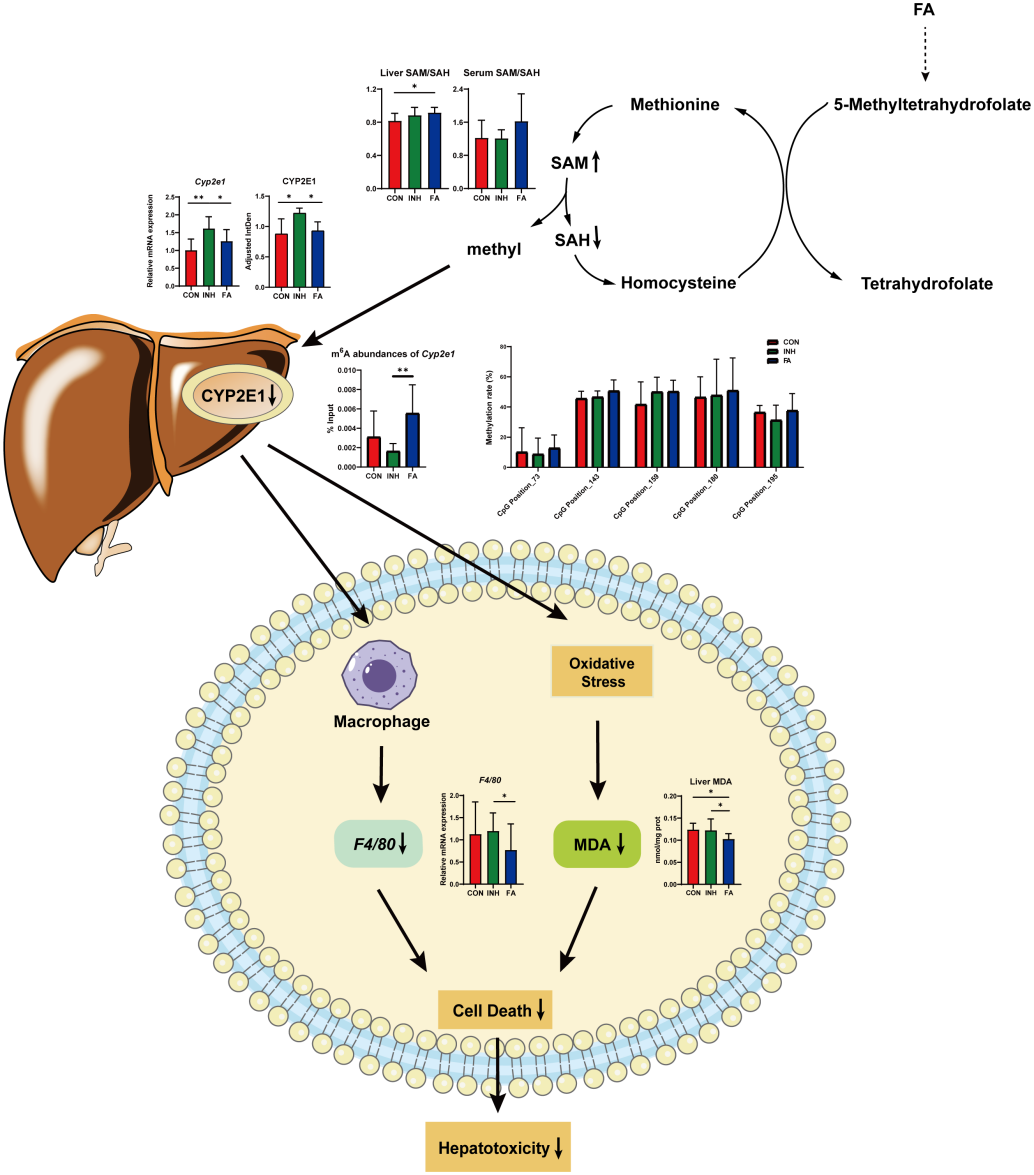
Diagram of the mechanism by which FA protects against INH-induced liver injury via the methylation of cytochrome P450 2E1. FA, folic acid; INH, isoniazid; SAM, S-adenosyl methionine; SAH, S-adenosyl homocysteine; CYP2E1, cytochrome P450 2E1; m^6^A: *N*^6^-methyladenosine; MDA, malondialdehyde; *F4/80*, mouse EGF-like module-containing mucin-like hormone receptor-like 1.

First, FA treatment alleviated INH-induced liver injury which may be related to its inhibition effect on CYP2E1 expressions. Our findings indicated that FA supplementation decreased the mRNA and protein expressions of CYP2E1, resulting in reduced liver MDA and *F4/80* mRNA expression and alleviated INH-induced liver injury (Figure 7). Elevated CYP2E1 expression is a characteristic feature in tuberculosis-drug-induced liver injury (26). Animal experiments showed that the extracts from *Rhus chinensis Mill*. and *Panax japonicus* can inhibit the expression of CYP2E1 to reduce oxidative stress and prevent liver injury (27, 28). In ethanol-fed pigs, FA deficiency led to the activation of hepatic CYP2E1, which promoted liver steatosis and apoptosis (29).

Second, FA supplementation may reduce the expression of CYP2E1 via enhancing RNA methylation. For RNA methylation, our results indicated that the m^6^A abundances of *Cyp2e1* were markedly decreased after INH treatment and restored after FA treatment. RNA m^6^A modification plays a critical role in regulating RNA stability, splicing, and translation, involving in the epigenetic regulation of drug-metabolizing enzymes (30). Previous study showed that 3-deazaadenosine treatment inhibited RNA methylation and led to a significant increase in P450 enzyme mRNA levels in HepaRG cells (31). The m^6^A modification is co-regulated by its methyltransferase and demethylase (32). Knockdown of m^6^A demethylase Fat Mass and Obesity-associated Protein (FTO), which increased the overall m^6^A level, inhibited the colony-forming ability of hepatocellular carcinoma cells and improved disease prognosis (33). The genes regulated by m^6^A methylation were related to the poor prognosis of hepatocellular carcinoma and the immune microenvironment, which was expected to become a new tool for evaluating the prognosis of patients with hepatocellular carcinoma (34). For DNA methylation, the methylation level of CpG island is important critical in the regulation of gene transcriptions (35). However, no CpG island was predicted on the *Cyp2e1* gene and the methylation level of five CpG sites were analyzed. Our results indicated no significant effect of FA on DNA methylation level of these CpG sites.

Third, FA supplementation may enhance RNA methylation levels via regulating the one-carbon cycle (Figure 7). Dietary and endogenous folate can regulate hepatic methionine metabolism and serve as a one-carbon source in DNA, RNA and protein methylation (36). In the liver, the elevation of SAH and the reductions of SAM and SAM/SAH were related with hypomethylation (37). In the current study, FA treatment increased the liver ratio of SAM/SAH and tend to increase the serum ratio of SAM/SAH. The liver and serum ratio tend to be positively correlated with the m^6^A abundance of *Cyp2e1*. Consistently, previous studies suggested that FA treatment significantly decreased the level of SAH and increased the level of SAM and the ratio of SAM/SAH in the ethanol-fed pig model to relieve liver injury (20). Another study indicated that folate deficiency significantly increased the level of SAH, decreased the level of SAM and SAM/SAH ratio, and aggravated the liver fibrosis in non-alcoholic liver injury mice (38).

In conclusion, FA treatment alleviated INH-induced liver injury which may be associated with its inhibition effect on CYP2E1 expressions through enhancing RNA methylation. Future study is warranted to further validate this mechanism.

## Data availability statement

The original contributions presented in the study are included in the article/supplementary material, further inquiries can be directed to the corresponding author.

## Ethics statement

The animal study was approved by Experimental Animal Welfare Ethics Committee of Qingdao University. The study was conducted in accordance with the local legislation and institutional requirements.

## Author contributions

LJ: Data curation, Investigation, Writing – original draft, Formal analysis. YN: Investigation, Writing – review & editing. CZ: Formal analysis, Validation, Writing – review & editing. DG: Investigation, Writing – review & editing. XG: Investigation, Writing – review & editing. KX: Funding acquisition, Supervision, Writing – review & editing. JW: Conceptualization, Funding acquisition, Supervision, Writing – review & editing, Formal analysis, Investigation.

## Footnotes

^1^ https://www.ncbi.nlm.nih.gov/gene
